# Unlocking the potential of microfluidics in mass spectrometry-based immunopeptidomics for tumor antigen discovery

**DOI:** 10.1016/j.crmeth.2023.100511

**Published:** 2023-06-26

**Authors:** Charlotte Stutzmann, Jiaxi Peng, Zhaoguan Wu, Christopher Savoie, Isabelle Sirois, Pierre Thibault, Aaron R. Wheeler, Etienne Caron

**Affiliations:** 1 CHU Sainte Justine Research Center, Montreal, QC, Canada; 2 Department of Chemistry, University of Toronto, Toronto, ON, Canada; 3 Institute for Research in Immunology and Cancer, University of Montreal, Montreal, QC, Canada; 4 Department of Chemistry, University of Montreal, Montreal, QC, Canada; 5 Donnelly Centre for Cellular and Biomolecular Research, University of Toronto, Toronto, ON, Canada; 6 Institute of Biomaterials and Biomedical Engineering, University of Toronto, Toronto, ON, Canada; 7 Department of Pathology and Cellular Biology, University of Montreal, Montreal, QC, Canada

**Keywords:** microfluidics, mass spectrometry, peptide, MHC, HLA, single-cell proteomics, immunopeptidomics

## Abstract

The identification of tumor-specific antigens (TSAs) is critical for developing effective cancer immunotherapies. Mass spectrometry (MS)-based immunopeptidomics has emerged as a powerful tool for identifying TSAs as physical molecules. However, current immunopeptidomics platforms face challenges in measuring low-abundance TSAs in a precise, sensitive, and reproducible manner from small needle-tissue biopsies (<1 mg). Inspired by recent advances in single-cell proteomics, microfluidics technology offers a promising solution to these limitations by providing improved isolation of human leukocyte antigen (HLA)-associated peptides with higher sensitivity. In this context, we highlight the challenges in sample preparation and the rationale for developing microfluidics technology in immunopeptidomics. Additionally, we provide an overview of promising microfluidic methods, including microchip pillar arrays, valved-based systems, droplet microfluidics, and digital microfluidics, and discuss the latest research on their application in MS-based immunopeptidomics and single-cell proteomics.

## Teaser

Sample preparation is a major bottleneck in immunopeptidomics. Inspired by recent developments in the field of single-cell proteomics, we discuss herein why and how microfluidics technology offers promising solutions to solve this important limitation in the immunopeptidomics field.

## Introduction

The field of tumor-specific antigen (TSA) discovery has grown rapidly in recent years, driven by the increasing interest in cancer immunotherapy as a treatment approach.^1^^,^^2^^,^^3^^,^^4^ TSAs presented at the cell surface by major histocompatibility complex (MHC) (human leukocyte antigen [HLA] in human) class I or class II molecules are unique to an individual’s tumor and are not found in healthy cells, making them ideal targets for cancer vaccines and immune-based therapies. These therapies aim to stimulate the patient’s immune system to recognize and attack the tumor cells, leading to improved clinical outcomes and reduced side effects compared with traditional treatments such as chemotherapy and radiation therapy.^5^^,^^6^^,^^7^

With the recent advances in cancer immunotherapies such as checkpoint inhibitors and chimeric antigen receptor (CAR) T cell therapy, direct identification of actionable TSAs has become a critical step in the development of new treatments.^8^^,^^9^ In this regard, mass spectrometry (MS)-based immunopeptidomics is a promising approach because it can directly determine the amino acid sequence of TSAs as physical molecules.^10^^,^^11^^,^^12^ In its simplest form, immunopeptidomics involves the isolation of HLA class I- and class II-associated peptides by immunoaffinity capture, peptide elution, and subsequent sequence identification by MS following database searches.^13^^,^^14^^,^^15^ Once TSAs have been identified, their immunogenicity and anti-tumor efficacy can be evaluated in pre-clinical and clinical studies.^16^^,^^17^ However, one major challenge in using this method is the sample preparation, i.e., the isolation of HLA-associated peptides with low absolute quantities, as it requires relatively large sample volumes, making it difficult to apply to minute amounts of clinically relevant biospecimens that are widely accessible in cohort studies and biobanks.^18^^,^^19^ Thus, the applicability of immunopeptidomics in basic and translational research remains relatively limited, and innovative methodologies are urgently needed to unlock its full potential.

Recently, microfluidics has been proposed as a method to improve the isolation of HLA proteins and their peptide ligands for tumor antigen discovery.^20^^,^^21^ Microfluidics is a field of study that deals with fluid flow in structures (typically formed by microfabrication) with at least one dimension in the range of microns (10^-6^ m).^22^^,^^23^^,^^24^^,^^25^ Microfluidic technologies are rapidly evolving and currently find wide applications in biomedical research, including for single-cell omics analyses.^26^^,^^27^^,^^28^^,^^29^^,^^30^^,^^31^^,^^32^ New microfluidic techniques have also gained significant interest in MS and proteomics due to the capability of those techniques to isolate and identify proteins at the single-cell level. Inspired by those recent advances in the field of proteomics, we aim to outline below some of the challenges facing sample preparation in immunopeptidomics workflows and explore how microfluidics can offer innovative solutions to drive progress forward.

## Challenges in immunopeptidomics

We highlight below the reasons why immunopeptidomics still requires relatively high sample inputs and explore potential explanations for variable and suboptimal peptide recovery across research groups in immunopeptidomics. This will facilitate the development of appropriate methodologies to address these challenges.

### Why immunopeptidomics still requires relatively high sample inputs?

#### The abundance of MHC-associated peptides is generally low

The abundance of cell surface MHC molecules can vary greatly between cell types and individuals, i.e., from 0 to >3,000,000 MHC molecules per cell (Table 1).^14^^,^^33^^,^^34^^,^^35^^,^^36^^,^^37^ Such variations affect the absolute quantity of peptides that are presented by MHC molecules. In general, the abundance of peptides presented by MHC molecules is low, typically ranging from ∼100 to ∼500 copies per cell, although extreme cases can go up to over 100,000 copies per cell (Table 2).^38^^,^^39^^,^^40^^,^^41^ Given this high heterogeneity, estimating the theoretical number of peptides that can be obtained from typical biopsies is a challenging task, as it depends on the expression level of MHC molecules and the distribution of peptide abundances in the immunopeptidome of each cell type present.

**Table 1:** Absolute quantification of cell surface MHC proteins (copies per cell) by flow cytometry

Species	Cell type	MHC/HLA	Quantification method	Number of MHC copies per cell (number of patients tested)	Reference
Human	B-ALL cells	HLA-A, -B, -C	QIFIKIT	∼550,000	Lanoix et al.^33^
Human	B-LCL cells	HLA-A, -B, -C	QIFIKIT	∼3,400,000	Lanoix et al.^33^
Human	JY cells	HLA-A, -B, -C	QIFIKIT	∼723,000	Sirois et al.^14^
Human	normal B cells	HLA-A, -B, -C	QIFIKIT	∼32,000–256,500 (7)	Kowalewski et al.^34^
Human	normal B cells	HLA-DR	QIFIKIT	∼19,500–79,500 (7)	Kowalewski et al.^34^
Human	CLL cells	HLA-A, -B, -C	QIFIKIT	∼42,500–288,500 (7)	Kowalewski et al.^34^
Human	CLL cells	HLA-DR	QIFIKIT	∼29,000–100,500 (7)	Kowalewski et al.^34^
Human	normal monocytes	HLA-A, -B, -C	QIFIKIT	∼75,300–239,500 (5)	Berlin et al.^35^
Human	normal monocytes	HLA-DR	QIFIKIT	0–3,250 (5)	Berlin et al.^35^
Human	AML blasts	HLA-A, -B, -C	QIFIKIT	∼45,000–262,000 (5)	Berlin et al.^35^
Human	AML blasts	HLA-DR	QIFIKIT	∼1,500–45,000 (5)	Berlin et al.^35^
Human	ovarian tumor cells	HLA-A, -B, -C	QIFIKIT	∼10,000–170,000 (11)	Schuster et al.^36^
Human	ovarian tumor cells	HLA-DR	QIFIKIT	∼20,000–300,000 (11)	Schuster et al.^36^
Human	endothelial cells	HLA-A, -B, -C	QIFIKIT	∼30,000–150,000 (11)	Schuster et al.^36^
Human	endothelial cells	HLA-DR	QIFIKIT	∼10,000–30,000 (11)	Schuster et al.^36^
Human	leukocytes	HLA-A, -B, -C	QIFIKIT	∼5,000–110,000 (11)	Schuster et al.^36^
Human	leukocytes	HLA-DR	QIFIKIT	0–45,000 (11)	Schuster et al.^36^
Mouse	B16F10 cells	H2-Db	QIFIKIT	∼2,700	Schuster et al.^37^
Mouse	B16F10 cells + IFNγ	H2-Db	QIFIKIT	∼288,000	Schuster et al.^37^
Mouse	B16F10 cells	H2-Kb	QIFIKIT	∼500	Schuster et al.^37^
Mouse	B16F10 cells + IFNγ	H2-Kb	QIFIKIT	∼90,000	Schuster et al.^37^
Mouse	LLC1 cells	H2-Db	QIFIKIT	∼14,500	Schuster et al.^37^
Mouse	LLC1 cells	H2-Kb	QIFIKIT	∼2,700	Schuster et al.^37^
Mouse	GL261 cells	H2-Db	QIFIKIT	∼67,000	Schuster et al.^37^
Mouse	GL261 cells	H2-Kb	QIFIKIT	∼28,700	Schuster et al.^37^
Mouse	EL4 cells	H2-Db	QIFIKIT	∼500,000	Schuster et al.^37^
Mouse	EL4 cells	H2-Kb	QIFIKIT	∼347,000	Schuster et al.^37^

QIFIKIT (quantitative analysis kit) was used to determine the number of MHC copies per cell for each cell type. B-ALL, B-LCL, and JY are EBV-transformed B cell lines. For primary normal or tumor cells obtained from patients, the number of patients for which MHC abundance was measured is mentioned in parentheses; the numbers indicate the range of MHC abundances measured across all patients for each cell type. Absolute abundance of cell surface MHC class I proteins was also measured in several mouse cell lines: B16F10 (melanoma cell line); LLC1 (Lewis lung carcinoma cell line); GL261 (glioblastoma cell line); and EL4 (lymphoma cell line). Original reference is indicated.

**Table 2:** Absolute quantification of MHC-associated peptides (copies per cell) by targeted MS

Peptide	MHC/HLA	Cell type	Targeted MS	Standard for quantitation (accuracy)	Number of peptide copies per cell	Reference
SLQDLIEKV	A∗02:01	SK-MEL-5 cells	PRM	hipMHC(+)	∼50	Stopfer et al.^38^
TLAEIAKVEL	A∗02:01	SK-MEL-5 cells	PRM	hipMHC(+)	∼50	Stopfer et al.^38^
GQVEIVTKV	A∗02:01	SK-MEL-5 cells	PRM	hipMHC(+)	∼20	Stopfer et al.^38^
KQVSDLISV	A∗02:01	SK-MEL-5 cells	PRM	hipMHC(+)	∼100	Stopfer et al.^38^
RTLAEIAKV	A∗02:01	SK-MEL-5 cells	PRM	hipMHC(+)	∼125	Stopfer et al.^38^
GLFDQHFRL	A∗02:01	SK-MEL-5 cells	PRM	hipMHC(+)	∼150	Stopfer et al.^38^
VLHDRIVSV	A∗02:01	SK-MEL-5 cells	PRM	hipMHC(+)	∼400	Stopfer et al.^38^
GVYDGEEHSV	A∗02:01	SK-MEL-5 cells	PRM	hipMHC(+)	∼500	Stopfer et al.^38^
KLADQYPHL	A∗02:01	SK-MEL-5 cells	PRM	hipMHC(+)	∼200	Stopfer et al.^38^
AMLGTHTMEV	A∗02:01	SK-MEL-5 cells	PRM	hipMHC(+)	∼150	Stopfer et al.^38^
SLYSYFQKV	A∗02:01	SK-MEL-5 cells	PRM	hipMHC(+)	∼250	Stopfer et al.^38^
KLDVGNAEV	A∗02:01	SK-MEL-5 cells	PRM	hipMHC(+)	∼800	Stopfer et al.^38^
SLADTNSLAVV	A∗02:01	SK-MEL-5 cells	PRM	hipMHC(+)	∼1,100	Stopfer et al.^38^
SLDDYNHLV	A∗02:01	SK-MEL-5 cells	PRM	hipMHC(+)	∼1,700	Stopfer et al.^38^
ALFDGDPHL	A∗02:01	SK-MEL-5 cells	PRM	hipMHC(+)	∼2,000	Stopfer et al.^38^
ALDGGNKHFL	A∗02:01	SK-MEL-5 cells	PRM	hipMHC(+)	∼10,000	Stopfer et al.^38^
HVDSTLLQV	A∗02:01	SK-MEL-5 cells	PRM	hipMHC(+)	∼3,500	Stopfer et al.^38^
RLLGTEFQV	A∗02:01	SK-MEL-5 cells	PRM	hipMHC(+)	∼40,000	Stopfer et al.^38^
RLLGTEFQV	A∗02:01	SK-MEL-5 cells +MEKi	PRM	hipMHC(+)	∼144,000	Stopfer et al.^38^
ALAPAPAEV	A∗02:01	JY cells	PRM	hipMHC(+)	∼390	Hassan et al.^39^
SLAADIPRL	A∗02:01	JY cells	PRM	hipMHC(+)	∼460	Hassan et al.^39^
VNYLHRNV	H2-Kb	EL4 cells	PRM	SIL(−)	∼910	Laumont et al.^40^
IILEFHSL	H2-Kb	EL4 cells	PRM	SIL(−)	∼5,000	Laumont et al.^40^
VTPVYQHL	H2-Kb	EL4 cells	PRM	SIL(−)	∼50	Laumont et al.^40^
TVPLNHNTL	H2-Db	EL4 cells	PRM	SIL(−)	∼18	Laumont et al.^40^
EEIPVSSHYF	B∗44:03	B-LCL cells	FAIMS-MS2	SIL(−)	∼115	Pfammatter et al.^41^
AEIQEKKEI	B∗44:03	B-LCL cells	FAIMS-MS2	SIL(−)	∼195	Pfammatter et al.^41^
AEIEQKIKEY	B∗44:03	B-LCL cells	FAIMS-MS2	SIL(−)	∼295	Pfammatter et al.^41^
EEIPVSSHY	B∗44:03	B-LCL cells	FAIMS-MS2	SIL(−)	∼257	Pfammatter et al.^41^
SEIEQKIKEY	B∗44:03	B-LCL cells	FAIMS-MS2	SIL(−)	∼182	Pfammatter et al.^41^
QELIGKKEY	B∗44:03	B-LCL cells	FAIMS-MS2	SIL(−)	∼100	Pfammatter et al.^41^
VEEADGNKQW	B∗44:03	B-LCL cells	FAIMS-MS2	SIL(−)	∼215	Pfammatter et al.^41^
SEESAVPKRSW	B∗44:03	B-LCL cells	FAIMS-MS2	SIL(−)	∼317	Pfammatter et al.^41^

Parallel reaction monitoring (PRM) and high-field asymmetric waveform ion mobility spectrometry (FAIMS)-MS2 were used for quantitative measurements of specific MHC I-associated peptides in different human and mouse cell lines: SK-MEL-5 (human melanoma cell line), JY and B-LCL (human EBV-transformed B cells), and EL4 (mouse lymphoma). Two formats of standards were used for absolute quantification: (1) heavy isotopically labeled peptide-MHCs (hipMHCs), which are more accurate (+), as they take into account peptide losses during immunoprecipitation, and (2) synthetic isotopically labeled (SIL) peptides, which are less accurate (−), as they do not take into account peptide losses during immunoprecipitation, thereby likely underestimating the number of peptide copies per cell (see Stopfer et al. for details^42^). Original reference is indicated. MEKi, MEK inhibitor binimetinib.

Experimental evidence has demonstrated the ability of MHC molecules to present highly immunogenic peptides, even at extremely low abundances, as low as a single copy per cell.^43^ To our knowledge, the detection limit for MHC-peptides using MS is typically in the attomoles range (50–500 amol), and assuming 100% purification efficiency, detecting a single molecule of a specific target peptide would require 30–300 × 10^6^ cells depending on the peptide sequence. This means that a typical tissue biopsy containing 1–10 × 10^6^ cells (1 mg) would not be adequate for detecting such target peptides using currently available MS methodologies.^44^^,^^45^^,^^46^ Moreover, if the peptide purification efficiency is reduced to 50%, the required cell number would double to 60–600 × 10^6^ cells. Therefore, relatively large number of cells (50–100 × 10^6^ cells)^47^^,^^48^ or tissue volume (5–200 mg tissue)^15^ are generally necessary to obtain sufficient amounts of peptides for immunopeptidomic analysis, depending on the cell type, MHC-peptide isolation protocol, and MS sensitivity. For the detection of rarer MHC-peptides, such as post-translationally modified (PTM) MHC-peptides, as much as 1 × 10^9^ cells can be required.^49^ This requirement is a major bottleneck. To overcome this limitation, several solutions have been proposed such as the expansion of tumor volumes in immunocompromised mice (patient-derived xenograft)^46^^,^^50^ or the amplification of single-cell patient material into clonal organoids.^51^^,^^52^ However, these solutions are not very efficient for rapid and systematic profiling of tumor immunopeptidomes in the clinic as it can be slow, expensive, impractical, and resource intensive and could potentially introduce experimental bias and confounders.

#### Peptide losses during the immunoaffinity purification procedure

**Figure 1:**
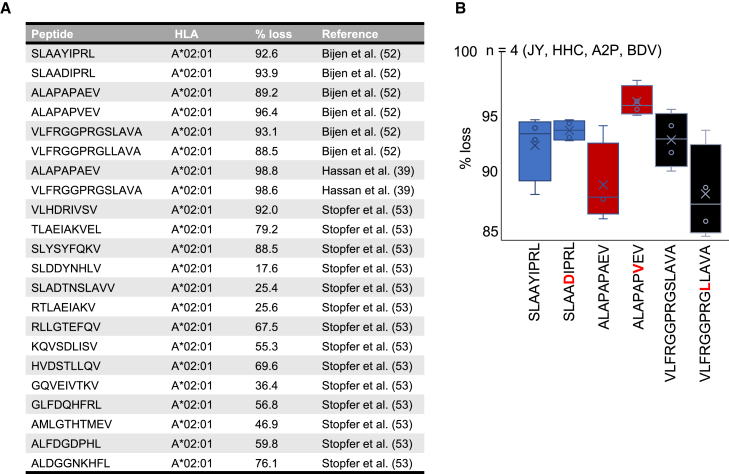
Peptide losses during the IP procedure (A) List of synthetic stable-isotope-labeled HLA peptide standards (MHC class I isotopolog calibrants) that were used in three independent immunopeptidomics studies. All the peptides bind HLA-A∗02:01. The proportion of HLA-A∗02:01 peptides that was lost during the traditional IP method is indicated for each peptide. The reference for each peptide is also indicated. (B) Proportion of peptide lost during the traditional IP method for 3 wild-type versus mutated peptides. Mutated amino acid is in red. Peptide loss was quantified for each peptide in four different lymphoblastoid cell lines: JY, HCC, A2P, and BDV.

Another reason that has recently become clear for the use of large sample inputs in immunopeptidomics is the relatively low yield of the immunoaffinity purification (IP) procedure for the isolation of MHC-peptides. This low yield, measured using specific reagents, reduces the sensitivity of downstream MS analysis, making high sample inputs necessary. The reagents used to accurately measure peptide losses during the IP procedure include heavy and medium synthetic stable-isotope-labeled HLA peptide standards (MHC class I isotopolog calibrants) and were recently described in several studies by the groups of van Veelen^39^^,^^53^ and White.^38^^,^^42^^,^^54^^,^^55^ Indeed, Hassan et al. were the first to apply these reagents by refolding recombinant HLA-A∗02:01 α chains and β2M with heavy peptides to generate heavy pHLAs (hpHLAs), which were then added to the cell lysate prior to IP as an embedded reference.^39^ The medium-labeled peptide was added exogenously to the sample prior to liquid chromatography with tandem MS (LC-MS/MS) analysis at the same concentration. Targeted MS analysis using parallel reaction monitoring (PRM) was then used to quantify pHLA losses by determining the ratio of the heavy to medium peptide signal. The results revealed striking losses in the IP and sample processing stages, ranging from 98.5% to 99.1% for the ALAPAPAEV peptide and from 97.2% to 99.5% for the VLFRGGPRGSLAVA peptide (average value shown in Figure 1A).^39^ In an additional study, the same group further investigated the yield of the IP procedure using six different peptides (3 wild-type and 3 mutated peptides) in four different lymphoblastoid cell lines (LCLs) for a total of 24 measurements.^53^ Consistently, their results showed substantial peptide losses between 85% and 98% within an average loss of 92% ± 4% (Figure 1A). Notably, their data indicated that even a single amino acid point mutation can significantly impact the yield of a peptide. This observation is evident by the differences observed in each pair of mutated and wild-type peptides across the four LCL conditions tested (Figure 1B). Then, the group of Forest M. White applied a similar approach to estimate peptide losses using 14 different hpHLA-A∗02:01.^38^^,^^42^ Their data showed that peptide losses ranged from 17.5% to 92% during the IP, with an average peptide loss of 56% ± 23% (Figure 1A). They also noted that there was no clear correlation between sample losses and peptide hydrophobicity or predicted peptide binding affinity to HLA-A∗02:01.^42^ Notably, the White and van Veelen laboratories used very different IP conditions, which are discussed below. Thus, the data currently available in the literature suggest that sample losses can be relatively substantial and vary depending on the peptide and experimental conditions.

### Reasons for variable and suboptimal peptide recovery

Delving into the reasons behind such substantial sample losses and behind variations between laboratories may help us understand how to mitigate losses and improve detection sensitivity and reproducibility.

#### Researchers utilize diverse IP conditions and purification methods

Many protocols have been optimized over the last 5 years for the isolation of MHC-associated peptides for their subsequent analysis by MS.^14^^,^^15^^,^^33^^,^^56^^,^^57^^,^^58^^,^^59^^,^^60^^,^^61^ Various IP conditions and purification methods are employed in those protocols, which may contribute to the differences in peptide recovery observed between laboratories such as those reported between the White and van Veelen labs. We highlight those differences below.

Research groups tend to use different ionic, non-ionic. or zwitterionic non-denaturing detergents for cell lysis and membrane disruption. Among the most widely used include 3-cholamidopropyl dimethylammonio 1-propanesulfonate (CHAPS), Zwittergent, Triton X-100 (Triton), Nonidet P-40 (NP40), detergent sodium deoxycholate (DOC), and IGEPAL CA-630 (Igepal).^62^ Those detergent are all compatible with immunopeptidomics workflows and do not create interference with peptide chromatography and detection. CHAPS is widely used and was shown to provide the highest peptide recovery when compared with Igepal, Triton, and DOC.^63^ On the other hand, Igepal was identified as a suboptimal detergent because it was shown to partially disrupt membranes, leading to the isolation of contaminant peptides during the IP process.^64^

The selection of the appropriate antibody is an important consideration that can influence the success of the experiment. The W6/32 antibody is established and widely used for the isolation of HLA-ABC-associated peptides. Other antibodies are less established and may lead to variations. For example, although both L243 and LB3.1 antibodies affinity capture HLA-DR, LB3.1 may yield at least double the number of MHC-peptides.^15^ Additionally, the amount of antibody used is a crucial factor that can vary among research groups and may impact the effectiveness of the IP. In this regard, the selected antibody needs to be in high excess for MHC depletion, as it has been observed that MHC is generally not depleted in the sample if low amounts are used, leading to low peptide recovery. To avoid this issue, a ratio of 10–50 μg antibody per 1 × 10^6^ cells (1–5 mg antibody per 1 × 10^8^ cells) is generally applied based on recent studies. Alternatively, the Strep-tag technology has been recently employed to study the mouse immunopeptidome of specific cell populations *in vivo.*^65^ The Strep-tag technology showed promising results in terms of sensitivity for the isolation of MHC-associated peptides and could potentially replace antibodies in engineered Strep-tag-MHC mouse models given their high efficiency for protein purification by affinity capture.^66^ A direct comparison between antibody and Strep-tag technology remains to be done.

Researchers also use different peptide elution and purification protocols. Immunocaptured MHC-associated peptides have to be eluted from Sepharose or magnetic beads. Elution is performed using 10% acetic acid^60^ or 1% TFA.^14^ Once MHC-peptide complexes have been eluted, an additional purification step is necessary to separate peptides from large proteins (antibody, β2-microglobulin, and MHC heavy chains), which would otherwise interfere with peptide chromatography and detection. Most commonly used separation techniques include reversed-phase high-performance LC (RP-HPLC),^15^ C18-solid phase extraction (C18-SPE),^14^^,^^60^ and 5–10 kDa cutoff filters,^54^^,^^56^^,^^67^ the latter two being the simplest and most widely used methods. Nevertheless, RP-HPLC was reported to provide the best peptide recovery when compared with C18-SPE and 5 kDa cut-off filtering.^63^

Regarding the yield evaluation experiments described above from the White and van Veelen laboratories, very different purification strategies were used. In the van Veelen laboratory, 2 × 10^9^ cells were lysed using Zwittergent and high amounts of antibody (17.5 mg antibody/sample) and large volumes of protein beads (7 mL) were used for IP. In the White laboratory, 200 times fewer cells (1 × 10^7^ cells) were lysed using CHAPS, and 0.2 mg antibody and 20 μL bead slurry (Fastflow protein A) were used for IP. Both groups eluted MHC-peptide complexes using 10% acetic acid followed by a 10 kDa cutoff filter for peptide separation. Notably, the antibody/cell ratio is >2-fold higher in the White laboratory, with 20 μg antibody/1 × 10^6^ cells compared with 8.75 μg antibody (Ab)/1 × 10^6^ cells in the van Veelen laboratory, which may have affected MHC depletion differently and could explain, among other factors, variations in peptide yields between the two groups.

#### Peptide adsorption on surfaces

It is known that sample losses in proteomics and peptidomics are caused by protein and peptide adsorption on surfaces used during the experiment, as well as the transfer between different containers. One of the main reasons for polypeptide adsorption is the “hydrophobic effect,” where strong interactions occur between the hydrophobic amino acid components of polypeptides and the hydrophobic surface of standard polymeric lab materials such as tubes and tips.^68^ Additionally, other adsorption mechanisms can also come into play, depending on the chemical characteristics of the polypeptides, such as their polarity, structure, charge, and size, which can enhance their affinity to polymeric surfaces and make the cause of sample losses more complex and untraceable.^69^^,^^70^ As the concentration of these molecules decreases, the loss of polypeptides in solution becomes more severe. Below a critical concentration, most peptides can be lost to adsorption, leaving nothing in solution to detect or analyze.^71^^,^^72^^,^^73^^,^^74^^,^^75^ The protocol used to purify MHC-associated peptides, which involves multiple steps with plastic surfaces,^14^^,^^15^ can exacerbate peptide adsorption, resulting in substantial losses of low-abundance MHC-associated peptides, including potentially valuable TSAs. Moreover, the use of organic solvent with plastic tubes can solubilize incomplete polymeric products that will contaminate peptide extracts potentially leading to undesired MS signal suppression effects.

Together, substantial and variable loss of peptides can be observed in immunopeptidomics, which can be attributed to a combination of factors, including the use of very different purification strategies as well as peptide adsorption on surfaces. New strategies are therefore needed to facilitate standardization of MHC-peptide isolation procedures from low-input samples while increasing peptide yield and reproducibly.

## Recent advances in microfluidics-based immunopeptidomics

Microfluidic devices can effectively reduce peptide adsorption by limiting the amount of peptides that can adsorb onto surfaces through their small channel dimensions and reduced surface area, as shown by numerous studies in MS-based proteomics.^76^^,^^77^^,^^78^^,^^79^^,^^80^^,^^81^^,^^82^^,^^83^^,^^84^^,^^85^^,^^86^^,^^87^^,^^88^^,^^89^^,^^90^^,^^91^ Microfluidic devices also operate with small sample volumes, which help reduce losses imparted to large surface contact areas. Moreover, the automatable characteristics of microfluidics can minimize human error and increase reproducibility of purification processes from ultra-low sample inputs. Furthermore, microfluidics has proven effective in isolating peptides^92^^,^^93^ and proteins by IP using antibodies,^81^^,^^94^ suggesting its potential for isolating HLA-peptide complexes and eluted peptides using similar methods. Thus, microfluidics is, in principle, a promising solution to isolate HLA-associated peptides more efficiently. If tested and validated, microfluidics could be applied to profile tumor immunopeptidomes from small amounts of clinical biospecimens without the need to expand them in patient-derived xenograft (PDX) models or organoid culture. Moreover, the development of robust and automated microfluidics technologies in immunopeptidomics could provide an opportunity to improve the throughput, quantitative accuracy, and accessibility of MHC-peptide measurements by MS.

Currently, only two microfluidics-based methods have been reported for isolating and analyzing HLAI-associated peptides using MS: PeptiCHIP^20^ and CHIP-IP.^21^ These methods were developed independently by two different research groups and both utilize microchips composed of thousands of micropillars, which are commonly used in microfluidic devices to improve target capturing efficiency.^95^^,^^96^

### The PeptiCHIP study

**Figure 2:**
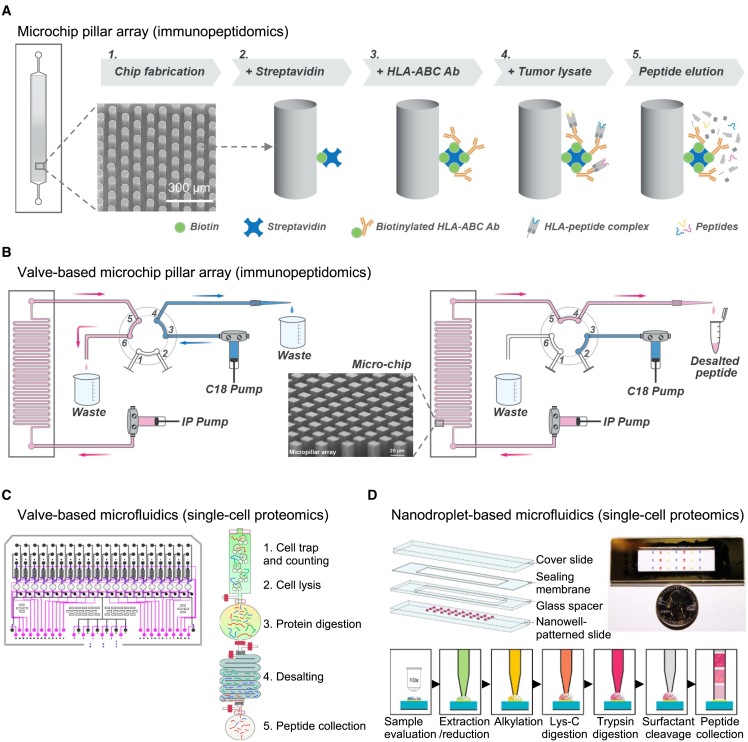
Microfluidics in immunopeptidomics and single-cell proteomics (A) PeptiCHIP is a microchip pillar array used as an immunopurification platform for immunopeptidomic applications. The PeptiCHIP is 30 mm long, 4 mm wide, and composed of 14,400 micropillars. A schematic overview describing the PeptiCHIP methodology is illustrated. Adapted from Feola et al., 2021.^20^ Copyright © 2021 the authors (https://creativecommons.org/licenses/by/4.0/). Published by American Chemical Society. See original publication for details.^20^ (B) CHIP-IP is a valve-based microchip pillar array for immunopeptidomics applications. The C18 cartridge and the CHIP-IP device were connected via an automated 6-port switch valve. The switch valve could be programmed to rotate to different positions, allowing for two operational modes (left and right). In the decoupled mode, the C18 cartridge was individually pre-conditioned while the IP was ongoing (left). In the coupled mode, the eluted peptides flowed directly into the conditioned C18 cartridge (right). Adapted from Li et al., 2023.^21^ Copyright © 2023 the authors (https://creativecommons.org/licenses/by/4.0/). See original publication for details.^21^ (C) Valve-based microfluidics for single-cell proteomics: (left) a schematic of the overall SciProChip layout. The control layer is shown in pink, while the flow layer is shown in black and blue. Note that SciProChip contains 20 operational units. (Right) Operational procedures of iProChip for streamlined sample preparation, including (1) cell trapping, imaging, and counting, (2) cell lysis, (3) protein digestion, (4) desalting, and (5) peptide collection. Adapted from Gebreyesus et al., 2022.^90^ Copyright © 2022 the authors (https://creativecommons.org/licenses/by/4.0/). See original publication for details.^90^ (D) Nanodroplet-based microfluidics: schematic drawing and photograph showing the nanoPOTS chip for conducting nanoproteomics studies from low cell numbers. Reprinted from Zhu et al., 2018.^86^ Copyright © 2018 the authors (https://creativecommons.org/licenses/by/4.0/). See original publication for details.^86^

In the first CHIP study, Vincenzo Cerullo and his team proposed a method for achieving IP of HLA-peptide complexes using a single 3-cm-long micropillar chip.^20^ Note that the chip’s physical design was not primarily tailored for the immunopeptidome enrichment workflow but rather for general proteomics applications, as mentioned.^96^ Specifically, the chip was comprised of 14,400 micropillars and was fabricated using a thiol-ene polymer and a UV-replica molding technique, resulting in a low-cost microfabrication process (Figure 2A). The chip was optimized with a layer height of 200 μm, a micropillar diameter of 50 μm, and a density of 100 μm interpillar distance, which allows for proper filling via capillary forces and minimizes the risk of blockages caused by bioaggregates and other particulate impurities.

For performing IP, the micropillars were functionalized with streptavidin-biotin and then coated with biotinylated pan-HLA-ABC antibody (W6/32 antibody) (Figure 2A). The PeptiCHIP was then used to trap HLA-I complexes by loading cell lysate directly onto it. Following washing, the complexes were removed by acid elution, and the process continued off chip with standard immunopeptidomics procedures, including purification of eluted HLA peptides with SepPac-C18 in acetonitrile.

While acknowledging the significance of this study, it is important to note several limitations that were identified. First, the strategy of using biotinylated antibodies contrasts with commonly used methods including association with protein A/G or crosslinking. Although a direct comparison was not performed by the authors, it is reasonable to suspect that antibody specificity and/or sensitivity could decrease, negating any benefits of the microfluidics platform. Second, according to the authors, their method enabled lower antibody consumption. For the in-vial-IP experiment, 10 mg antibody was used for 1 × 10^9^ cells, whereas for the in-PeptiCHIP experiment, they used 45 μg antibody for 4.5 × 10^6^ cells. While it is correct that the PeptiCHIP required less antibody, the ratio of antibody per cell remained constant at 10 μg per 1 × 10^6^ cells. Third, the use of the SepPac-C18 cartridge may appear counterproductive to the goal of achieving greater sensitivity since the smallest cartridge available is larger than what is typically used for microfluidics-based sample preparation. This could potentially explain why the number of peptides identified using this method does not appear significantly different from those identified using conventional immunopeptidomics sample preparation techniques. For instance, from 1 × 10^6^ cells, 1,804 peptides were identified, of which 67% were predicted to be good HLA binders (Table 3). Fourthly, purification of HLAI-associated peptides using PeptiCHIP led to atypical contamination of long peptides (above 10-mer) and a low HLA-I binder content. Finally, the PeptiCHIP workflow exhibited poor system integration, as the fluidic control was not adequately demonstrated.

**Table 3:** Performance and comparison of multiple microfluidic technologies for the isolation and analysis of HLA-bound peptides (immunopeptidomics) or proteins (proteomics) by MS

Immunopeptidomics					
Cell type	No. cells	No. peptides detected	HLA-I binders	Microfluidics	Reference
JY (B cell line)	10 × 10^6^	2,100 1,134	82% 82%	PeptiCHIP Vial-IP	Feola et al.^20^
JY (B cell line)	1 × 10^6^	1,804 387	67% 41%	PeptiCHIP Vial-IP	Feola et al.^20^
Ovarian tumor	10 mg 60mg	172 1,128	ND ND	PeptiCHIP PeptiCHIP	Feola et al.^20^
Bladder cancer (patient organoid)	6 × 10^6^	2,089	ND	PeptiCHIP	Feola et al.^20^
ccRCC (patient organoid)	6 × 10^6^	576	ND	PeptiCHIP	Feola et al.^20^
RA957 (B cell line)	10 × 10^6^ 2 × 10^5^	15,000 4,000	>90% >85%	CHIP-IP CHIP-IP	Li et al.^21^
RA957 (B cell line)	1 × 10^6^	10,000 5,100	>90% >90%	CHIP-IP Vial-IP	Li et al.^21^
Melanoma tumor	5 mg 40 mg	7,149 13,724	>95% >95%	CHIP-IP CHIP-IP	Li et al.^21^

Proteomics				
Cell type	No. cells	No. proteins detected	Microfluidics	Reference
293T (human embryonic kidney cells)	4 13 68 119	913 1,563 2,271 2,770	valve-based continuous flow (Online Rare Cell Separation [ORCS] proteomics)	Wang et al.^91^
MCF7 (breast epithelial cells)	61	2,000	valve-based continuous flow (ORCS proteomics)	Wang et al.^91^
Circulating tumor cells from patients	5–7	973–1,135	valve-based continuous flow (ORCS proteomics)	Wang et al.^91^
HeLa (human epithelial cells)	10–141 10–140	3,092–3,460 313–2,048	droplet microfluidics (NanoPots) Vials	Zhu et al.^86^
HeLa (human epithelial cells)	21–93	1,763–2,260	droplet microfluidics (μPots)	Xu et al.^88^
Mouse liver tissue	10–160	1,275–2,077	droplet microfluidics (μPots)	Xu et al.^88^
*C. elegans*	959	4,698 4,442	digital microfluidics (DropBot) Vials	Steinbach et al.^82^
PC-9 (lung adenocarcinoma cells)	5–106	1,638–4,722	valve-based continuous flow (iProChip)	Gebreyesus et al.^90^
MEC-1 (chronic B cell leukemia)	1–117	455–3,811	valve-based continuous flow (iProChip)	Gebreyesus et al.^90^
PC-9 (lung adenocarcinoma cells)	1	1,500	valve-based continuous flow (SciProChip)	Gebreyesus et al.^90^
U87 (glioblastoma cells)	1	427	digital microfluidics (digital microfluidic isolation of single cells for omics [DISCO])	Lamanna et al.^80^

ND, not determined.

Despite the above limitations, the PeptiCHIP was effective in isolating and identifying HLA-associated peptides from patient-derived material such as ovarian tumors, bladder tumors, and clear cell renal cell carcinoma (ccRCC) (Table 3). Some of those peptides were shown to elicit a CD8^+^ T cell response. Thus, the PeptiCHIP method has obvious limitations and needs further optimization but represents a stepping stone toward the creation of more advanced microfluidics-based techniques in immunopeptidomics.

### The CHIP-IP study

The CHIP-IP platform was specifically designed to fulfill the unique requirements of the immunopeptidome enrichment workflow.^21^ The CHIP-IP boasts a serpentine-curved fluidic microchannel measuring 50 cm in total length, as illustrated in Figure 2B. The microchannel comprises roughly 250,000 micropillars, each with a side length of 20 μm and a height of 100 μm. The workflow necessitates a relatively low sample volume (100 μL) and utilizes an automated fluidic control system, which integrates C18 cartridges required for sample cleanup via a programmable switch valve (Figure 2B). This attribute is important in immunopeptidomics, as it eliminates the need for unnecessary sample transfers, resulting in enhanced assay sensitivity. Moreover, clamping reinforcements were incorporated into the CHIP-IP to bolster its mechanical robustness, preventing any leakage or sample loss.

Using the RA957 B cell line, the authors conducted a direct comparison of peptide recovery between the CHIP-IP and the traditional in-vial-IP methods. An extensive RA957-specific spectral library was built in Spectronaut, and immunopeptidomics data were acquired in data-independent acquisition (DIA) mode to boost sensitivity, as described.^97^ CHIP-IP resulted in approximately twice the number of identified peptides compared with the in-vial-IP method when only 1 × 10^6^ cells were used (Table 3). Specifically, ∼10,000 and ∼5,100 HLAI-associated peptides were identified using CHIP-IP and vial-IP, respectively (Table 3), thereby indicating that sample preparation was a bottleneck for achieving high peptide recovery when working with low sample input. Notably, >4,000 and >7,000 HLAI-associated peptides were identified from 2 × 10^5^ RA957 cells and 5 mg melanoma tissue, respectively. To fully assess the capabilities of CHIP-IP, it will be important to conduct tests on a broader range of cell and tissue types encompassing varying levels of HLA abundance. This comprehensive evaluation will provide valuable insights into the versatility and effectiveness of the CHIP-IP method across different biological contexts.

Peptide recovery is affected by IP conditions, as described above. In the CHIP-IP method, micropillars were coated with protein A before loading them with the W6/32 antibodies, which were subsequently crosslinked. The CHIP-IP was loaded with 300 μg antibody, irrespective of the starting amount of cells used for IP. For example, 300 μg antibody per 10 × 10^6^ cells represents a ratio of 30 μg antibody per 1 × 10^6^ cells, which fall within the conventional 10–50 μg antibody per 1 × 10^6^ cells generally used. However, 300 μg antibody per 2 × 10^5^ cells results in an excessively high antibody-per-cell ratio, 50 times greater than the conventional ratio. This ratio likely ensures complete capturing of HLAI-peptide complexes in the sample and potentially enhances peptide recovery from low sample inputs. In the future, it will be important to use heavy isotopically labeled peptide-MHC (hipMHC) standards (as shown in Table 2) for accurate yield measurements while developing and testing new microfluidic devices and IP conditions.

Although CHIP-IP has shown promising results, its accessibility needs improvement due to the complexity of its tubing and pump systems, as well as the requirement for expertise in microengineering and cleanroom operation. Nonetheless, the technology represents an important milestone in the field, as it lays the groundwork for the automation and commercialization of sensitive devices that can facilitate robust clinical immunopeptidomics.

### Can antibody-free CHIP methods be a better solution for quantitative immunopeptidomics?

Robust and accurate quantification of therapeutically relevant cell surface TSAs from low sample inputs could be pivotal in defining the threshold of positive responsiveness to immunotherapies, such as antibody-drug conjugates, cancer vaccines, bispecific T cell engagers, or other T cell-based therapies.^98^ Therefore, the development of simple antibody-free methods for eluting cell surface MHC-peptides and their integration into microfluidics could significantly advance our understanding of immunopeptidomes and their implications for disease treatment.

Mild acid elution (MAE) is a straightforward antibody-free method that offers an alternative to IP for eluting cell surface MHC class I-associated peptides from intact cells.^33^^,^^56^ Although MAE has been successfully applied to resuspended cells,^99^^,^^100^^,^^101^^,^^102^^,^^103^^,^^104^ its use for solid tissues was deemed impractical due to the high proportion of contaminant peptides, and the method is no longer utilized in the field. However, with the advent of innovative microfluidics technologies and the ability to work with smaller numbers of resuspended cells in a CHIP, MAE has the potential to be revisited as a valuable method for quantitative measurements of MHC-associated peptides using targeted MS. As the peptides to be targeted by MS are already known, they do not need to be in a highly pure MHC-peptide pool for effective detection and quantification.

Moreover, while IP isolates both intracellular and cell surface MHC-peptides, MAE isolates peptides from the cell surface, which is the most immunologically relevant peptide pool for T cells. DIA-MS, together with the use of pre-established MHC-peptide spectral libraries, could be used to perform high-throughput targeting of cell surface MHC-peptides for relative quantification of immunopeptidomes across different conditions.^97^^,^^105^^,^^106^^,^^107^ Additionally, PRM could be used with hipMHC to provide absolute quantification of a subset of clinically relevant MHC-peptides. This pre-defined set of peptides may include PTM MHC-peptides in autoimmunity^108^^,^^109^^,^^110^ or TSA in cancer,^111^^,^^112^ and applying a relatively simpler antibody-free CHIP method to elute them could represent a significant advantage to measure rapidly, accurately, and reproducibly their cell surface abundance over time in the context of a longitudinal study, for instance.

Furthermore, the integration of an antibody-free MHC-peptide elution workflow into a microfluidics device would facilitate its scalability and accessibility to other laboratories. Therefore, the development of such methods, while not perfect at providing pure MHC-peptide pools, may be of great complementary value for the field. The emerging field of single-cell proteomics could provide insights to accelerate progress toward this direction.^113^

## Insights from single-cell proteomics

Advanced proteomics techniques have been developed to enable the analysis of small sample sizes (<1,000 cells) using MS, greatly expanding the scope of proteomic analysis.^114^^,^^115^^,^^116^^,^^117^^,^^118^ Recently, new valve-based continuous flow microfluidics,^79^^,^^90^^,^^91^ nanodroplet microfluidics,^86^^,^^88^^,^^89^ and digital microfluidics^80^^,^^82^^,^^83^ approaches were reported to enhance proteome profiling sensitivity, even from a single cell (Table 3). The following is a brief overview of those approaches, which can provide valuable insight for further microfluidics development in immunopeptidomics.

### Valve-based continuous flow microfluidics

Valve-based continuous flow microfluidics is a type of microfluidics technology that utilizes tiny valves and pumps to control the flow of liquids through microscale channels (Figure 2C). This approach allows for precise control over the flow rate and direction of fluids, which can be adjusted in real time.^119^ Several valve-based microfluidics systems have been developed for proteomics analysis over the last years.^79^^,^^90^^,^^91^ Recently, two advanced valved-based microfluidic devices, the iProChip and the SciProChip, were developed for the analysis of <100 cells and for the analysis of single cells, respectively.^90^ A great feature of these devices is the integration of cell capture and counting, cell lysis, protein digestion, and desalting in an integrated microfluidic workflow, thus reducing sample loss due to multistep transfer. Specifically, the analytical performance and versatility of iProCHIP were demonstrated using the PC-9 cell line (human adenocarcinoma cells) and the MEC-1 cell line (chronic B cell leukemia cells) (Table 3). The results showed that the dynamic range of protein abundance spans 5 orders of magnitude, a wide quantification range (>100-fold) over which accurate quantification was possible for specific proteins of interest, good reproducibility (Pearson correlation of 0.88–0.98), and low missing values (<16%) between runs. Additionally, the SciProChip was able to detect 1,500 ± 131 protein groups (false discovery rate [FDR] 1%) using DIA-MS from a single PC-9 cell, making it one of the most sensitive methods for analyzing the proteome of a single mammalian cell (Table 3). In immunopeptidomics, a similar valved-based microfluidic system has not been developed yet. However, it is anticipated that this system could be further microengineered and combined with micropillar arrays coated with pan-HLA antibodies to control the flow and direction of fluids, resulting in highly specific and more sensitive identification of HLA-associated peptides.

### Nanodroplet-based microfluidics

In proteomics, nanodroplet-based microfluidics is a technique that involves handling extremely small volumes of fluid, typically on the nanoliter scale within an immiscible medium, such as oil droplets suspended in water, or vice versa.^87^ This approach allows for highly efficient and precise processing of trace amounts of protein samples, enabling in-depth proteome analysis with minimal sample loss.^87^ For instance, the group of Ryan T. Kelly reported a robotically addressed chip-based nanodroplet processing platform for enhancing proteomic sample processing and analysis from a small cell population. The platform, which is known as nanodroplet processing in one pot for trace samples (nanoPOTS), reduces total processing volumes from the conventional hundreds of microliters to <200 nL within a single droplet reactor (Figure 2D).^86^ When coupled with highly sensitive MS, nanoPOTS enabled reproducible proteomic measurements of >3,000 proteins from as few as ∼10 HeLa cells (Table 3). NanoPOTS is a powerful platform, but its reliance on a costly, in-house-built robotic nanopipetting instrument and associated expertise may limit its dissemination to a broader research community. To address this limitation, the same group has developed μPOTS^88^ and autoPOTS,^120^ a more accessible version of the method that uses a commercially available micropipette and a commercially available robot for liquid handling, respectively. This study shows the capability of μPOTS to accurately identify a large number of proteins from a small number of cells, including ∼1,800 proteins from ∼25 HeLa cells and ∼1,200 proteins from ∼10 mouse liver cells (Table 3).

The potential application of low-microliter droplets in IP of specific protein complexes is an area that remains largely unexplored, but the concept shows great promise. The isolation of HLA-peptide complexes is one potential application by combining the advantages of the nanodroplet and surface modification, and if successful, this approach could be adapted by researchers in the field for ultra-sensitive immunopeptidomics.

Another potential avenue for exploration is adapting the MAE protocol to the NanoPots system. In this approach, intact living cells could be attached to the nanowells and treated with MAE, and eluted peptides from the cell surfaces could then be separated and analyzed through an adapted chromatographic system. Although this method may be less specific, peptides of interest, pre-determined by DDA-MS, could be targeted by PRM or DIA-MS for quantitative analysis across different samples.

Furthermore, the recent success of the NanoPots system in performing imaging MS for mapping the spatial distribution of proteins across tissue surfaces suggests that it could be adapted for quantitative measurement of HLA-peptides.^121^^,^^122^ Such an adaptation could pave the way for the emergence of spatial immunopeptidomics, which would be particularly informative for understanding the heterogeneity of immunopeptidomes *in vivo*.^106^^,^^123^

### Digital microfluidics

**Figure 3:**
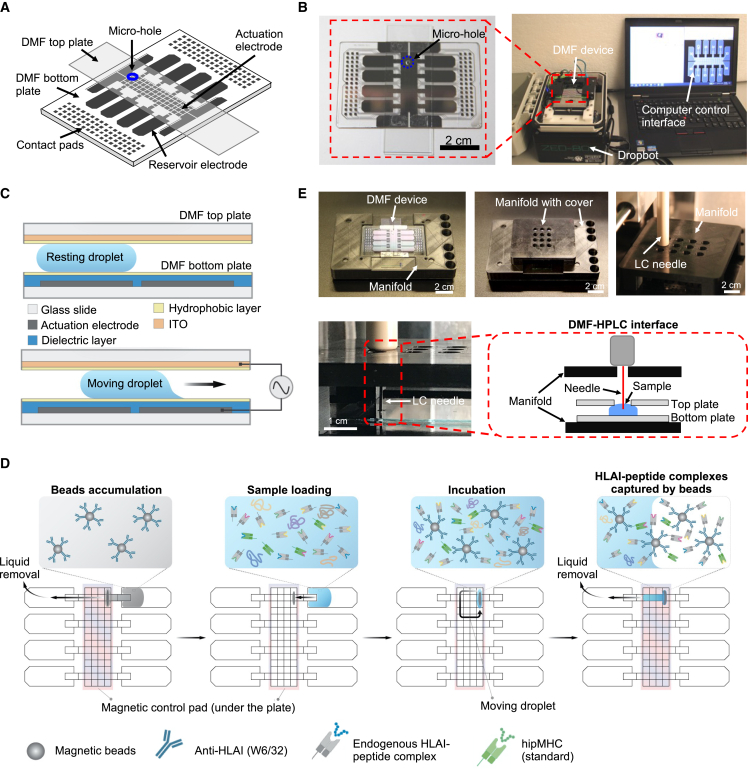
All-in-one digital microfluidics (DMF) pipeline for proteomic and immunopeptidomic sample processing and analysis (A) Cartoon of the DMF device, including a bottom plate and a top plate featuring a sampling microhole (blue). (B) Photograph of a DMF device interfaced with the open-source DropBot control system and computer running the open-source MicroDrop program. (C) Schematics of DMF device (side view) illustrating the components of the DMF device, including glass substrates (white), actuation electrodes on the bottom plate (gray), the dielectric layer on the bottom plate (blue), the hydrophobic layers (yellow), and the indium tin oxide (ITO) counter-electrode on the top plate (orange). When no electric potential is applied (top), the droplet is immobile. When an electric potential is applied to a particular driving electrode (bottom), the droplet moves onto the electrode. (D) This schematic illustrates a DMF device designed to enrich HLAI-peptide complexes. In this hypothetical scenario, magnetic beads cross-linked with the W6/32 antibody are first introduced into the device and accumulate in a specific location. Next, the sample containing HLAI-peptide complexes is loaded onto the device, and during the incubation period, the complexes are captured by the W6/32 antibody-coated magnetic beads. This results in the enrichment of HLAI-peptide complexes, which can then be used for downstream purification steps. (E) Photographs of customized DMF-autosampler manifold without (top left) and with (top middle) the custom cover. (Top right) Photograph of the top cover of the manifold bearing sampling array holes that support the autosampler injector needle. (Bottom) Photograph and schematic (inset) illustrating the sampling process in the DMF-HPLC interface. (A)–(C) and (E) were reproduced from Peng et al., 2023.^134^ with permission from the Royal Society of Chemistry. See original publication for details.^134^

Digital microfluidics (DMF) is a technology that enables precise manipulation of small amounts of liquid using an electrode array and the principle of electrowetting on dielectric (EWOD)^124^^,^^125^ (Figures 3A–3C). One of its key advantages is the ability to control individual droplets of liquid, ranging from several microliters down to nanoliters, without the need for micropipettes^126^ (Figure 3C). The technology ensures precise spatial isolation, preventing any unwanted mixing of fluids. Additionally, DMF is a pump-free technology that eliminates the need for a complex tubing network, making it easier to implement and disseminate across laboratories (Figure 3B). To date, DMF has been widely used in biomedical research and has demonstrated success in various applications.^127^^,^^128^^,^^129^^,^^130^^,^^131^^,^^132^^,^^133^ In proteomics, it has been utilized to analyze proteomes from as few as 100 cells^82^^,^^83^^,^^84^ and even single cells using the DMF isolation of single cells for omics (DISCO) technique^80^ (Table 3).

DMF has already shown potentials for use in immunopeptidomics, having been used for the IP of proteins prior to MS analysis.^81^ There is potential for further development of this technology for IP of HLA-peptide complexes for the analysis of tumor immunopeptidomes. For instance, DMF could be tested for the enrichment of HLA-peptide complexes using antibody-coupled magnetic beads (Figure 3D). By placing a magnetic control pad underneath the DMF device, the beads could be easily manipulated. Peptides could also be potentially captured and eluted within the DMF device using C18-SPE magnetic beads, as described.^135^

To further reduce sample loss, Peng et al. recently described a DMF-powered “all-in-one pipeline” for proteomic sample processing and analysis. The pipeline is an end-to-end integrated process, including an automated interface to LC with MS (DMF-HPLC-MS interface) (Figure 3E), and may serve as a base to build on for the proposed immunopeptidomics workflow. If tested and validated, this method could be widely adopted in the immunopeptidomics community using, for example, the open-source DropBot platform^136^ (Figure 3B), ultimately leading to its commercialization for robust and sensitive TSA profiling in the clinical setting.

In addition to advancements in sample preparation, the implementation of new MS detection techniques, such as timsTOF SCP, can significantly enhance sensitivity and provide more comprehensive data for immunopeptidomes.^137^ Moreover, cutting-edge computational tools based on machine learning, such as MS2Rescore,^138^ Prosit,^139^ and AlphaPeptDeep,^140^ can assist with MS/MS prediction and rescoring. These tools are particularly advantageous for detecting low-input samples and have the potential to substantially enhance the accuracy of immunopeptidome detection and quantification.

### Conclusion

The field of immunopeptidomics is faced with a significant analytical challenge relating to the recovery of HLA-associated peptides and TSAs during sample preparation, in addition to its inherent low quantities in biological samples. This is due to sample losses on container walls and other surfaces during the multiple pipetting steps required for the IP of HLA-associated peptides. Microfluidics has been proposed as a solution to this issue, as it can reduce fluid contact with surfaces, enable automation of sample preparation steps, and integrate with MS. However, to date, only two studies have demonstrated the feasibility of using microfluidics to profile tumor immunopeptidomes. Therefore, the development and application of microfluidics technologies in immunopeptidomics is still in its early stages, thus providing an opportunity for multidisciplinary collaborations and innovations. Some existing microfluidic application scenarios for low-input or single-cell samples, such as on-chip cell sorting, cell lysis, protein purification, digestion, labeling, and desalting, may have the potential to be adapted and used for the isolation, enrichment, purification, and identification of HLA-associated peptides. Given the importance of TSAs in cancer immunotherapy, the development of new microfluidics devices and technologies in future immunopeptidomics is expected to become an active area of research in the coming years.
